# Multi-frequency VEMPs improve detection of present otolith responses in bilateral vestibulopathy

**DOI:** 10.3389/fneur.2024.1336848

**Published:** 2024-02-21

**Authors:** F. Lucieer, M. van der Lubbe, L. van Stiphout, M. Janssen, V. Van Rompaey, E. Devocht, A. Perez-Fornos, N. Guinand, R. van de Berg

**Affiliations:** ^1^Division of Balance Disorders, Department of Otorhinolaryngology, Head and Neck Surgery, Maastricht University Medical Centre, Maastricht, Netherlands; ^2^Department of Methodology and Statistics, Care and Public Health Research Institute, Maastricht University, Maastricht, Netherlands; ^3^Department of Otorhinolaryngology and Head and Neck Surgery, Antwerp University Hospital, Faculty of Medicine and Health Sciences, University of Antwerp, Antwerp, Belgium; ^4^Service of Otorhinolaryngology Head and Neck Surgery, Department of Clinical Neurosciences, Geneva University Hospitals, Geneva, Switzerland

**Keywords:** bilateral vestibulopathy (BV), vestibular evoked myogenic potential (VEMP), multi-frequency, vestibular, cVEMP, OVEMPs, otolith

## Abstract

**Objective:**

To investigate whether multi-frequency Vestibular Evoked Myogenic Potential (VEMP) testing at 500, 750, 1,000, and 2,000 Hz, would improve the detection of present dynamic otolith responses in patients with bilateral vestibulopathy (BV).

**Methods:**

Prospective study in a tertiary referral center. BV patients underwent multi-frequency VEMP testing. Cervical VEMPs and ocular VEMPs were recorded with the Neuro-Audio system (v2010, Neurosoft, Ivanovo, Russia). The stimuli included air-conducted tone bursts of 500, 750, 1,000, and 2,000 Hz, at a stimulation rate of 13 Hz. Outcome measures included the percentage of present and absent VEMP responses, and VEMP thresholds. Outcomes were compared between frequencies and type of VEMPs (cVEMPs, oVEMPs). VEMP outcomes obtained with the 500 Hz stimulus, were also compared to normative values obtained in healthy subjects.

**Results:**

Forty-nine BV patients completed VEMP testing: 47 patients completed cVEMP testing and 48 patients completed oVEMP testing. Six to 15 % more present VEMP responses were obtained with multifrequency testing, compared to only testing at 500 Hz. The 2,000 Hz stimulus elicited significantly fewer present cVEMP responses (right and left ears) and oVEMP responses (right ears) compared to the other frequencies (*p* ≤ 0.044). Using multi-frequency testing, 78% of BV patients demonstrated at least one present VEMP response in at least one ear. In 46% a present VEMP response was found bilaterally. BV patients demonstrated a significantly higher percentage of absent VEMP responses and significantly higher VEMP thresholds than healthy subjects, when corrected for age (*p* ≤ 0.002). Based on these results, a pragmatic VEMP testing paradigm is proposed, taking into account multi-frequency VEMP testing.

**Conclusion:**

Multi-frequency VEMP testing improves the detection rate of present otolith responses in BV patients. Therefore, multi-frequency VEMPs should be considered when evaluation of (residual) otolith function is indicated.

## Introduction

Bilateral vestibulopathy (BV) is a chronic disorder in which the vestibular function is bilaterally severely reduced or absent (1). It has many etiologies, varying from toxic (e.g., gentamicin ototoxicity), infectious (e.g., meningitis) and genetic (e.g., DFNA9), to inner ear disease (e.g., Menière’s disease, auto-immune inner ear disease) and neurodegenerative disease (e.g., CANVAS) (2). BV patients report a spectrum of symptoms, of which chronic unsteadiness and/or oscillopsia are most frequently reported (3, 4). These symptoms often result in a decreased quality of life and a high socio-economic burden on society (4, 5). BV can be diagnosed using the diagnostic criteria of the Bárány Society. These criteria include a chronic vestibular syndrome with symptoms of unsteadiness when walking or standing (possibly combined with oscillopsia), and a bilaterally reduced or absent angular vestibulo-ocular reflex function. This latter should be documented by at least one of the following three tests: video Head Impulse Test, caloric test or rotatory chair test (6). Currently, only lateral semicircular canal function is included in the diagnostic criteria of BV, not otolith function. This implies that in patients diagnosed with BV, otolith function can still be present (7–9).

Vestibular evoked myogenic potentials (VEMPs) measure dynamic otolith function by stimulating the Type I hair cells at the striola (10, 11). VEMPs are electromyographic responses to air-conducted sound or bone-conducted vibration of the skull, which most likely reflect otolith function. Two types of VEMPs can be measured: cervical VEMPs (cVEMP) and ocular VEMPs (oVEMP). Cervical VEMPs comprise inhibitory responses from the ipsilateral sternocleidomastoid muscle, mainly evaluating function of the saccule. Ocular VEMPs comprise excitatory responses from the contralateral inferior oblique extra-ocular muscle, mainly evaluating function of the utricle. The testing paradigm and interpretation of VEMPs are difficult to standardize (12). Since VEMP response characteristics (amplitude, latency, threshold) depend on, e.g., stimulus type, muscle-contraction and age, each laboratory should obtain its own normative data. However, even after correcting for differences in muscle contraction, variability in VEMPs can be large in normal subjects (13).

In BV, VEMPs vary widely. BV can lead to reduced or absent VEMP responses, but in a significant number of patients VEMPs are within the normal range (7–9). The number of reduced, absent or present VEMPs in BV patients differs between studies. This most likely reflects the heterogeneity of testing paradigms, outcome measures and patient populations used in these studies (7). For example, some etiologies like aminoglycoside toxicity might be associated with otolith abnormalities (14). Moreover, it remains difficult to perfectly understand VEMPs in BV due to the large variability in normal subjects. After all, VEMPs might be reduced due to BV, but still be within the “broad” normal range, leading to false negative results (7). Additionally, age significantly affects VEMPs, resulting in a high rate of absent responses in normal subjects above the age of 60 years (12). Since the age of the BV population is relatively high (8), an absent VEMP response in a BV patient might reflect age, BV, testing paradigm, or a combination of these factors. In these patients, the influence of BV in the observed VEMP responses remains unknown. Furthermore, as stated above, otolith function is not included in the diagnostic criteria of BV. Patients with disorders predominantly affecting otolith function, might therefore be missed and not included in BV studies (7, 15, 16).

It was previously found that different acoustic stimulus frequencies, evoke different VEMP responses (17). For example, cVEMP responses in the affected ears of patients with Menière’s disease, demonstrate a significantly higher cVEMP threshold at tone bursts of 500 Hz than at 1,000 Hz, compared to normal subjects (18, 19). This implies that “frequency tuning” exists in the vestibular system. This “frequency tuning” is also affected by age. In young normal adults, the largest VEMP responses are obtained around 500 Hz. In older adults (≥ 60 years), the largest VEMP responses are more often obtained at 750 and 1,000 Hz in the majority of cases. It was therefore recommended to test 750 and 1,000 Hz tone burst frequencies, in case absent responses are found at 500 Hz (20).

In the last decades, a novel treatment was proposed to treat BV: the vestibular implant. This is a (not yet clinically available) strategy to partially restore vestibular function by stimulating the vestibular nerves, using surgically implanted electrodes (21–23). The electrodes can be implanted inside or close to the semicircular canals (24, 25), or inside the otolith organs (26). Surgically positioning electrodes in a semicircular canal or otolith organ, can destroy the (residual) function of that specific organ. Consequently, otolith implantation is currently only considered in case of bilaterally absent cVEMP and oVEMP responses (27). In BV subjects it is therefore imperative to understand, before considering vestibular implantation, whether otolith function is present or not. However, previous VEMP studies in BV patients mainly tested at 500 Hz (8, 9, 14). This might imply that some BV patients that were considered to have absent VEMP responses, might have had preserved VEMPs at other test frequencies.

The objective of this study was therefore to investigate whether multi-frequency VEMP testing (500, 750, 1,000, and 2,000 Hz) would improve the detection of present otolith responses in BV patients.

## Methods

### Patient population

The patient population was previously described (4, 28–33). In short, BV patients who were previously diagnosed according to the diagnostic criteria of the Bárány Society (6) at Maastricht University Medical Center, were included in this prospective study. Adult BV patients were invited for a testing day that also involved examinations related to other BV studies (4, 28–33). BV patients who were not able to undergo the detailed audiovestibular testing, or who did not want to talk about one of the investigated topics (e.g., psychological symptoms), or who were not able to stop vestibulo-suppressive medication, were excluded from this study.

### VEMP testing

Cervical VEMPs and oVEMPs were recorded with the Neuro-Audio system (v2010, Neurosoft, Ivanovo, Russia) and self-adhesive electrodes (Blue sensor, Ambu, Denmark). For cVEMPs, the recording electrodes were placed on the sternocleidomastoid muscles and the reference electrode on the sternum. For oVEMPs, the recording electrodes were placed on the orbital margin inferior to both eyes, and the reference electrode approximately 2 cm below them. For both c-and oVEMPS, the ground electrode was placed on the forehead (8). The order of testing was randomized for VEMP type (cVEMP and oVEMP) and for stimulation side (right ear and left ear).

Cervical VEMPs were measured in supine position. The head was flexed 30° and turned away from the stimulation side. A monitor (v2010, Neurosoft, Ivanovo, Russia) provided visual feedback to the patient regarding sternocleidomastoid muscle contraction. Patients were instructed to control muscle contraction between 65 and 205 μV. This was indicated on the monitor as a meter which should be held in a green area. Red areas on the monitor indicated contractions which were too low or too high. Two-hundred electromyography traces with muscle contraction between 65 and 205 μV were required (8). Cervical VEMPs were elicited with air-conducted tone bursts, provided by inserted earphones. The stimuli included air-conducted tone bursts of 500, 750, 1,000, and 2,000 Hz. A Blackman gating window was used with a two cycle rise/fall without a plateau. The resulting rise time was 4.00 ms at 500 Hz, 2.66 ms at 750 Hz, 2.00 ms at 1,000 Hz, and 1.00 ms at 2,000 Hz. Thirteen Hertz was chosen as stimulation rate, to decrease testing time (34). Furthermore, no significant difference was found regarding present and absent cVEMP and oVEMP responses, when comparing normative VEMP data of 5 and 13 Hz (500 Hz air-conducted tone bursts) obtained in our vestibular laboratory (*p* ≥ 0.063).

Ocular VEMPs were also measured in supine position. Patients were instructed to keep their eyes fixed on a target which was located 30 degrees behind their head on the ceiling of the examination room. This achieved superomedial gaze. The same stimulus parameters were used as for cVEMPs, but for oVEMPS a minimum of 300 electromyography traces were required (8).

A staircase approach was adopted to determine VEMP thresholds. Steps of 5 dB SPL were used, which started at 130 dB SPL. The threshold was defined as the lowest sound level that elicited detectable P1 and N1 peaks. A trial repetition was performed to confirm the absence of P1 and N1 peaks at the sound level just below threshold (8). Stimulation was not corrected for conductive hearing loss, since no significant conductive hearing loss was present in any of the patients, as tested by audiometry (Interacoustics Affinity audiometer and Easidata software). All tested ears demonstrated ≤20 dB air-bone gaps at all tested frequencies. The median air-bone gaps of right and left ears separately, were 5 dB for each tested frequency.

To obtain normative data for our vestibular laboratory, normative cVEMP data was obtained in 51 healthy subjects (29 women, mean age 47 years, standard deviation 20 years). Normative oVEMP data was obtained in 48 healthy subjects (27 women, mean age 49 years, standard deviation 19 years).

### Statistical analysis

Data were analyzed using SPSS Statistics 28 for Windows. VEMP outcome measures included the percentage of present and absent VEMP responses, and VEMP thresholds. A VEMP response was considered present, in case a response could be obtained (regardless of the absolute threshold). A VEMP response was considered absent, in case no response could be obtained at the highest stimulus level. Regarding thresholds, the sound level (dB SPL) was used as input for the statistical analysis. In case of an absent response, a (hypothetical) sound level of 140 dB SPL was used. This number was chosen to facilitate conservative calculations, since it was very close to the highest tested sound level (130 dB SPL).

The Cochran’s *Q* test was used to determine whether the proportion of patients who had a VEMP response differed across the 4 stimulus frequencies. In case of a significant Cochran’s Q test, *post hoc* paired analyses were carried out using multiple McNemar’s tests. Since every BV patient was tested at multiple frequencies, the assumption of independence was violated. Therefore, the relationship between stimulus frequency (500, 750, 1,000, and 2,000 Hz) and VEMP sound level threshold (dB SPL) was investigated using marginal linear regression analyses with unstructured covariance matrix of the residuals. The effect of stimulus frequency was adjusted for ear (left, right), age, gender and starting side of the threshold measurements (left, right ear). In addition, to test for a possible differential effect, the interaction between frequency and ear was first included in the model and removed again if it was not significant (top-down strategy). Linear regression analysis was applied to compare the mean threshold levels (dB SPL) between BV patients and healthy controls adjusted for age. To compare the BV patients and controls with respect to the occurrence of a present VEMP response after correction for age, logistic regression was performed. Mean differences in threshold levels and odds ratio’s for no present VEMP response were reported as BV patients compared to healthy controls. The α-value was set to 0.05. In case of multiple comparisons, Bonferroni correction was applied. Two-sided Bonferroni corrected (exact) *p*-values were reported, unless stated otherwise.

### Ethical considerations

This study was performed in accordance with the Declaration of Helsinki (amended version 2013). Approval was obtained from the ethical committee of Maastricht University Medical.

Center (NL52768.068.15/METC). All subjects provided written informed consent.

## Results

### Patient characteristics

Forty-nine BV patients underwent multi-frequency VEMP testing in this study. This included 24 women (49%). Mean age of all patients was 60 years (minimum 21 years, maximum 79 years). Etiologies included ototoxicity (22%), infectious (16%), genetic (14%), Menière’s disease (6%), metabolic (4%) and auto-immune disease (2%). The etiology in approximately 35% of patients remained idiopathic. Forty-seven patients completed cVEMP testing and 48 patients completed oVEMP testing. In total, multi-frequency cVEMPs and oVEMPs could both be obtained in 46 patients. Reasons for not completing multi-frequency VEMP testing in all patients included: tiredness (*n* = 1), neck pain (*n* = 1) and equipment failure (*n* = 1).

### Presence of multi-frequency VEMP responses in BV patients

**Figure 1:**
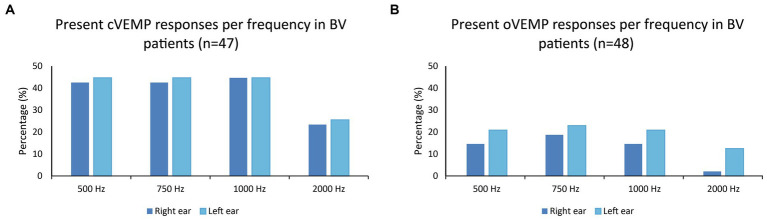
**(A,B)** Percentages of present cVEMP and oVEMP responses in BV patients, classified by stimulus frequency (500, 750, 1,000, and 2,000 Hz).

Figures 1A,B present the percentages of present cVEMP and oVEMP responses in BV patients, classified by stimulus frequency. Regarding cVEMPs, it can be observed that a present cVEMP was found in more than 40% of the patients for the frequencies 500 to 1,000 Hz. This was significantly lower for 2,000 Hz compared to the other frequencies (right ears: *p* ≤ 0.009; left ears: *p* = 0.018). Marginal regression analysis showed that cervical VEMP thresholds were significantly higher at 2,000 Hz in both ears of the BV patients (*p* < 0.001). Regarding oVEMPs, the percentage of present VEMP responses was significantly lower for all frequencies compared to cVEMP in right ears (one-sided *p*-values ≤ 0.012). This was not the case for all frequencies in left ears: *p* = 0.040 (500 Hz), *p* = 0.012 (750 Hz), *p* = 0.052 (1,000 Hz), *p* = 0.360 (2,000 Hz). Present oVEMP responses were found in 15–23% for the frequencies 500 Hz to 1,000 Hz, and in less than 13% for 2,000 Hz (Figure 1B). Two-thousand Hertz elicited significantly fewer present oVEMP responses compared to the other frequencies in the right ears (*p* ≤ 0.044), but not in the left ears. However, marginal regression analysis did not show a significant relationship between the stimulation frequency and the oVEMP threshold values in both ears. The frequencies eliciting present VEMP responses differed between patients: not all patients with, e.g., a present VEMP response at 500 Hz, also showed a present VEMP response at 750 Hz. Patients with at least one present VEMP response, demonstrated (on average) present VEMP responses at 3.0 and 2.7 frequencies for cVEMP (right and left ears respectively), and at 2.4 and 2.3 frequencies for oVEMP (right and left ears respectively). The percentage of patients with at least one present VEMP response was therefore higher than the percentages found in Figure 1 (see also Table 1).

**Table 1:** Present VEMP responses: multi-frequency vs. only the 500 Hz stimulus in BV patients.

	cVEMP (*n* = 47)		oVEMP (*n* = 48)	
	*Multi-frequency*	*500 Hz*	*Multi-frequency*	*500 Hz*
Present response(s): right ear	51%	43%	21%	15%
Present response(s): left ear	60%	45% ^*^	33%	21% ^*^
Present response(s): at least one ear	70%	57% ^*^	35%	23% ^*^
Present response(s): both ears	40%	30% ^*^	19%	13%
All tested frequencies present (500–2,000 Hz)	15%	N/A	2%	N/A

Multifrequency VEMP response(s) are classified as present, in case at least one tested frequency demonstrated a present response. Multi-frequency included VEMP testing at 500, 750, 1,000, and 2,000 Hz. N/A, not applicable. ^*^Significant difference between multi-frequency testing and only testing at 500 Hz (one-sided value of *p* < 0.05).

### Detection of present otolith function in BV: multi-frequency vs. 500 Hz stimulus

Table 1 illustrates that, in the same BV population, more present otolith responses were found when using multi-frequency stimuli compared to only testing at 500 Hz. This was significant for cVEMP and oVEMP responses obtained in left ears, but not in right ears (Table 1). The difference in present responses between multi-frequency and 500 Hz stimulation could increase up to 15% when considering each ear separately (cVEMPs, left ears). Additionally, 70% of patients demonstrated a cVEMP response in at least one ear, and 35% of patients demonstrated an oVEMP response in at least one ear (Table 1). A bilaterally present VEMP response was found in 40% (cVEMP) and 19% (oVEMP) of the patients.

In some ears, a VEMP response was found at only one frequency, despite multifrequency testing (Table 2). This was the case in the minority of ears and did not always involve 500 Hz. For example, in 6% of the left ears, a cVEMP response was only present at 1,000 Hz.

**Table 2:** Multi-frequency testing: percentage of ears with a VEMP response at only one frequency.

	cVEMP (*n* = 47), only present response at:				oVEMP (*n* = 48), only present response at:			
	500 Hz	750 Hz	1,000 Hz	2,000 Hz	500 Hz	750 Hz	1,000 Hz	2,000 Hz
Present response: right ear	4%	0%	2%	0%	2%	2%	0%	0%
Present response: left ear	4%	4%	6%	0%	4%	2%	0%	2%

### Multi-frequency VEMPS: cVEMPs and oVEMPs combined

Forty-six BV patients completed both cVEMP and oVEMP testing, sufficient for analysis. Seventy-eight percent of these patients showed at least one present cVEMP or oVEMP response, in at least one ear. It was found that in 46% of BV patients, bilateral VEMP responses were found in cVEMP, or oVEMP, or both. However, this latter was only the case in 15% of the patients (Table 3).

**Table 3:** Bilaterally present multi-frequency VEMP responses (*n* = 46).

	Percentage of BV patients
Present bilateral responses: cVEMP	41%
Present bilateral responses: oVEMP	20%
Present bilateral responses: cVEMP or oVEMP or both	46%
Present bilateral responses: cVEMP and oVEMP	15%

Multifrequency VEMP response(s) are classified as present, in case at least one tested frequency demonstrated a present response. Multi-frequency included VEMP testing at 500, 750, 1,000, and 2,000 Hz.

### VEMP responses (500 Hz stimulus): BV patients vs. healthy subjects

VEMP responses (500 Hz stimulus) of the BV patients were compared to VEMP responses of healthy subjects. Cervical VEMP responses (right and left ears) were significantly more absent in BV patients compared to healthy subjects (Odds Ratio ≥ 4.462, *p* ≤ 0.002). Furthermore, mean thresholds of cVEMP responses (right and left ears) were significantly different in BV patients compared to healthy subjects (mean difference BV patients compared to healthy controls of ≥7.698 dB SPL, *p* ≤ 0.001). Ocular VEMP responses (right and left ears) were also significantly more absent in BV patients compared to healthy subjects (Odds Ratio ≥ 8.885, *p* < 0.001). The mean thresholds of oVEMP responses in BV patients compared to healthy controls (right and left ears) were significantly different as well (mean difference BV patients compared to healthy controls of ≥9.379 dB SPL, *p* < 0.001). In summary, BV patients demonstrated a significantly higher percentage of absent VEMP responses and significantly higher VEMP thresholds than healthy subjects, when corrected for age.

## Discussion

This study investigated whether multi-frequency VEMP testing (500, 750, 1,000, and 2,000 Hz) would improve the detection of present otolith responses in BV patients, compared to only testing at 500 Hz. It was demonstrated that more present otolith responses were obtained with multi-frequency testing. Present cVEMPs were more often present than present oVEMPs. Most present VEMP responses were found when testing at 500, 750, and 1,000 Hz, while 2,000 Hz resulted in fewer present VEMP responses and (for cVEMPs) significantly higher thresholds. These results show that multi-frequency VEMP testing should be considered in BV patients, in case evaluation of (residual) otolith function is indicated.

Multi-frequency VEMP testing improves the detection of present VEMP responses in BV patients, emphasizing the need to test “beyond” the 500 Hz stimulus. These findings are congruent with previous literature in healthy subjects, in which it was illustrated that 500 Hz is not always the best frequency for VEMP testing (20). This results from “frequency-tuning” of the vestibular system, in which different acoustic stimulus frequencies evoke different VEMP responses. Frequency-tuning is patient specific, since it might depend on multiple factors. These factors include, among others: etiology, stage of disease, age, and stimulation paradigm (18, 20). Since multi-frequency VEMP testing improves the detection of present VEMP responses in BV patients, it might be somehow analogous to ice water caloric testing (1). After all, multi-frequency VEMPs and ice water calorics can both be used to detect residual function in case no responses are obtained in the “routine tests” (respectively 500 Hz VEMPs and bithermal caloric testing). Furthermore, this study showed that fewer present VEMP responses and (for cVEMPs) significantly higher thresholds were found when testing at 2,000 Hz. This was expected, since in healthy subjects 2,000 Hz has also less robust responses and significantly higher thresholds than the other tested frequencies (35). This might not directly imply that VEMP testing at 2,000 Hz should be abandoned completely: one ear only demonstrated a 2,000 Hz oVEMP response, without any responses at the other tested frequencies (oVEMPs and cVEMPs). Future research should be conducted to investigate whether testing at other frequencies is beneficial.

Present VEMP responses were frequent in this BV population. Seventy-eight percent demonstrated at least one present VEMP response in at least one ear, and in 46% a present VEMP response was found bilaterally. A high percentage of present VEMP responses in BV patients is consistent with previous studies. However, direct comparison is difficult because of different BV populations, stimulation paradigms and outcome measures. Nevertheless, these results illustrate two important aspects. First, BV is currently a diagnosis based on lateral semicircular canal function: it does not include vertical semicircular canal function, or otolith function as measured by VEMPs (6, 9, 14). This implies that patients with predominantly affected vertical semicircular canal and/or otolith function, might be missed (7, 15, 16, 36). However, diagnosing (isolated) otolith dysfunction is still challenging. The clinical presentation of otolith dysfunction is not yet well understood and no consensus has been reached on this possible clinical entity (37–39). After all, absent VEMP responses are not necessarily causally related to vestibular symptoms, and do not rule out involvement of other structures (37). It would therefore be advised to further investigate the possible clinical entity of otolith dysfunction (38). Secondly, this study shows that VEMP responses are often still present in BV patients. On-the-one-hand this may imply that otolith function is relatively spared in BV patients compared to lateral semicircular canal function. It could be hypothesized that, e.g., otoliths are less affected by certain vestibular disorders. On-the-other-hand it might have nothing to do with otoliths being less affected than semicircular canals, but with VEMP testing itself. It could be hypothesized that VEMPs are relatively stronger stimuli to the otoliths than, e.g., bithermal caloric testing or video head impulse testing to the semicircular canals. In other words: although otolith function might be affected, a response remains present due to the strong nature of the stimulus. As a result, a present VEMP response is obtained, while hypofunction of the lateral semicircular canals is detected by the caloric test and/or video head impulse test. Additionally, VEMPs are considered to test Type 1 hair cells, while the caloric test might mainly test Type 2 hair cells (10). A dissociation between these tests could therefore also result from a difference in affected hair cell type. Furthermore, the interpretation of the tests may play a role. VEMPs are currently not able to detect subtle changes in otolith function, even with good normative data (7). This results from the large range of normal responses in healthy subjects (7). Therefore, except for superior semicircular canal dehiscence syndrome (40), VEMPs are clinically often interpreted as an “on–off” response: response are considered present or absent (27). It is however not justified to compare tests with different outcomes: categorical “on–off” (VEMPs) vs. numerical (e.g., caloric test slow phase eye velocities, video-head impulse test vestibulo-ocular reflex gain). For example, in cases with reduced (but still minimally present) otolith and lateral semicircular canal function, a reduced but present VEMP response might be obtained. This would then be classified as “normal.” The numerical outcomes of the caloric test and/or video head impulse would indicate a reduced function. These tests would then be classified as “abnormal.” However, if the caloric test and video head impulse test would be interpreted based on just the presence of a response, these tests would have also been classified as “normal” (e.g., a video head impulse test gain of 0.4 is still a response). Therefore, the dissociation between otolith and lateral semicircular canal findings in BV patients, should be interpreted with care. It might even be incorrect to state that, e.g., otolith function would be less affected than lateral semicircular canal function in BV patients.

Nevertheless, this study demonstrates that the diagnostic criteria of bilateral vestibulopathy should be revised in the future. A more detailed classification could be considered, taking into account all 10 vestibular sensors, not only the lateral semicircular canals. This might facilitate a detailed classification. For example, categories could vary from abnormal vestibular responses in all vestibular sensors, to isolated abnormal responses such as selective vertical canal or otolith impairment (10, 36).

In this study, the presence of present cVEMP responses was higher than present oVEMP responses. Air-conducted cVEMPs are therefore superior to air-conducted oVEMPs to detect present VEMP responses in BV patients. This does not necessarily indicate that, e.g., saccular function is less affected than utricular function. After all, these findings are not consistent with previous literature, in which saccular and utricular function were almost equally affected (14). Most likely, this can mainly be attributed to the stimulation paradigm: air-conducted sound (this study) vs. bone conducted vibration. Bone conducted vibration produces more reliable oVEMP responses than air-conducted sound (12). This implies that the oVEMP findings in this study probably underestimate the presence of present oVEMP responses in BV patients. It was aimed to use bone-conducted vibration in this study, but long lasting equipment failure unfortunately prevented this study from using bone-conducted vibration. Nevertheless, the objective of this study was to investigate the application of multi-frequency stimulation. This could still be accomplished, but these findings are only applicable to air-conducted stimulation.

**Figure 2:**
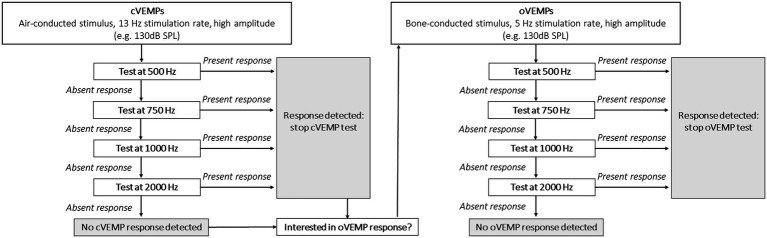
Proposal for a clinical VEMP testing paradigm in BV patients, if the clinician would focus on the presence or absence of a VEMP response.

BV patients demonstrated a significantly higher percentage of absent VEMP responses and significantly higher VEMP thresholds than healthy subjects, when corrected for age. This shows that VEMP responses can be significantly affected in BV patients. Unfortunately, as stated above, the large range of normal responses in healthy subjects does not allow to detect subtle changes in otolith function (7, 12). Therefore, VEMPs are mainly interpreted as an “on–off” response in BV patients (27). Taking these limitations and the results of this study into account, a pragmatic VEMP testing paradigm could be proposed for BV patients, if the clinician would only be interested in the presence or absence of a VEMP response. This paradigm is illustrated in Figure 2.

Its concept focuses on obtaining a relatively quick insight in VEMP responses: start testing at a high sound pressure level (e.g., 130 dB SPL) to maximize the detection rate; use a 13 Hz stimulus rate to minimize testing time (cVEMPs); use multi-frequency testing to improve detection rate; stop when one frequency shows a present response since presence of a VEMP response has been demonstrated. It should be noted that testing at 2,000 Hz may be optional. After all, only one ear demonstrated a present VEMP response at 2,000 Hz, without any responses at the other frequencies (Table 2: oVEMP, left ear). The proposed frequencies in this paradigm are based on this study, and other frequencies (e.g., 1,500 Hz) were not tested. Therefore, other frequencies could also be included. Additionally, based on previous literature it would be proposed to use a bone-conducted stimulus for oVEMP testing (12). However, the reliability of a 13 Hz stimulus for a bone-conducted stimulus should still be determined and therefore a 5 Hz stimulus would still be preferred for oVEMPs.

### Limitations

BV is a heterogeneous disorder and VEMP responses depend on many factors (e.g., age). This implies that findings of this study are restricted to this specific study population.

## Conclusion

Multi-frequency VEMP testing improves the detection rate of present otolith responses in BV patients. Therefore, multi-frequency VEMPs should be considered when evaluation of (residual) otolith function is indicated.

## Data availability statement

The raw data supporting the conclusions of this article will be made available by the authors, without undue reservation.

## Ethics statement

The studies involving humans were approved by Ethical Committee of Maastricht University Medical Center (NL52768.068.15/METC). The studies were conducted in accordance with the local legislation and institutional requirements. The participants provided their written informed consent to participate in this study.

## Author contributions

FL: Conceptualization, Investigation, Methodology, Visualization, Writing – original draft, Writing – review & editing, Data curation, Formal analysis, Project administration, Resources. ML: Investigation, Writing – review & editing, Formal analysis. LS: Writing – review & editing. MJ: Formal analysis, Methodology, Writing – review & editing. VR: Writing – review & editing. ED: Writing – review & editing. AP-F: Writing – review & editing. NG: Writing – review & editing. RB: Conceptualization, Data curation, Formal analysis, Funding acquisition, Methodology, Supervision, Validation, Visualization, Writing – original draft, Writing – review & editing.
